# Supplementary material for: Vitamin D deficiency and severity of pneumonia in Indonesian children
Source: PLoS One. 2021 Jul 9;16(7):e0254488. doi: 10.1371/journal.pone.0254488 (PMC8270442; doi:10.1371/journal.pone.0254488)
Supplement: S1 File — (PDF) [file pone.0254488.s001.pdf]

CASE REPORT FORM – Clinical data

The Burden of Pneumonia In Indonesia Children And The Association With Vitamin D Deficiency: The Indonesian Pneumonia And Vitamin D Study (I-PADS)

Participant's ID [ ][ ] / [ ][ ][ ][ ] /[ ][ ][ ] / I-PADS

<u>Instruction:</u> cross the box or complete with appropriate answer

Patient's name ____

Medical record number [ ][ ][ ][ ][ ][ ]

Sex 1. Male 2. Female

Date of Birth [ ][ ] / [ ][ ] / [ ][ ][ ][ ] (dd/mm/yyyy)

Admission date [ ][ ] / [ ][ ] / [ ][ ][ ][ ] ; time : [ ][ ]. [ ][ ]

Doctor on duty ___________________________________

1. Admission 1 [_] Policlinic 2 [_] Emergency Room
2. Referral case 1 [_] Yes 2 [_] No
   If yes, referred from: 1 [_] Primary Health Care Center 2 [_] Other Hospital
   3 [_] Physician/pediatrician private clinic 4 [_] Midwife private Clinic
3. Receive antibiotics before admission: 1[_] Yes 2[_] No 3[_] Not Known

4. Signs (Anamnesis)

Cough	1. Yes	2. No	Wheezing	1. Yes	2. No
Runny nose	1. Yes	2. No	Dyspnea	1. Yes	2. No
Fever	1. Yes	2. No	Noisy breathing	1. Yes	2. No
Fast breathing	1. Yes	2. No	Others, ______	1. Yes	2. No

5. Danger sign

Unable to drink/feed	1 [_] Yes	2 [_] No
Stridor	1 [_] Yes	2 [_] No
Lethargic/ unconscious	1 [_] Yes	2 [_] No
Cyanosis	1 [_] Yes	2 [_] No
Severe Respiratory distress	1 [_] Yes	2 [_] No
Seizure	1 [_] Yes	2 [_] No

6. Physical examination (at admission or during first 24 hours medical care)

Respiratory rate: [_][_][_] / minute Heart rate: [_][_][_] / minute axilla temp.: [_][_],[_]

Body weight: [_][_], [_] kgs Body weight/Length: [_][_][_], [_] cms

Chest indrawing (subcostal) 1 [_] Yes 2 [_] No

Wheezing 1 [_] Ada 2 [_] No

Pulse oximetry 1 [_] Performed 2 [_] Not Performed

If performed, Result SaO2: [_][_][_] % (without receiving oxygen)

7. Initial diagnosis:
Primary diagnosis
1 [_] Pneumonia 2[_] Asthma attack 3 [_] Bronchiolitis
4 [_] Others, mention:…………………………………
Secondary diagnosis
1 [_] Acute diarrhea 2[_] Sepsis
4 [_] Underweight 4 [_] Others: ……………………………….

8. B20:
1 [_] Yes 2[_] No 3 [_] Not examined

Participant's ID [ ][ ] / [ ][ ][ ][ ] /[ ][ ][ ] / I-PADS

COMPLETED BY RESEARCH ASSISTANT
Participant's name: ________________________________

9. Vitamin D level (completed by research assistant): [_][_][_] nmol
10. Blood examination

Hemoglobin	[ ][ ] , [ ][ ]	g/dL	Leucocyte	[ ][ ] , [ ][ ]	10^3 / µL
Hematocrit	[ ][ ] , [ ][ ]	%	Limphocyte	[ ][ ] , [ ][ ]	%
Erythrocyte	[ ][ ] , [ ][ ]	10^6 / µL	Neutrophil	[ ][ ] , [ ][ ]	%
MCV	[ ][ ] , [ ][ ]	fL	Eosinophil	[ ][ ] , [ ][ ]	%
MCH	[ ][ ] , [ ][ ]	pg	Basophil	[ ][ ] , [ ][ ]	%
MCHC	[ ][ ] , [ ][ ]	g/dL	Monocyte	[ ][ ] , [ ][ ]	%
Thrombocyte	[ ][ ][ ][ ][ ][ ]	/µL			

11. Radiology examination (Thorax) 1 [_] Performed 2[_] Not performed
Radiology result's conclusion from site (hospital):

	Yes	No
Normal		
Infiltrat		
Consolidation		
Milier		

	Yes	No
Atelectasis		
Hilar limphadenopathy		
Effusion		
Others:		

Conclusion:____________________

12. Initial antibiotics therapy given at admission in the hospital:

	Yes	No
Cotrimoxazole		
Amoxicillin		
Erithromycin		
Ampicillin		
Gentamycin		
Chloramphenicol		

	Yes	No
Cefotaxim		
Ceftriaxon		
Amoxicillin Clavulanat		
Penicillin		
Others, mention:		

13. B2 agonis via nebulizer 1 [_] Yes 2 [_] No
14. Steroid 1 [_] Yes 2 [_] No
15. Oral Salbutamol 1 [_] Yes 2 [_] No
16. Oxygenation 1 [_] Yes 2 [_] No
Start date [ ][ ] / [ ][ ] / [ ][ ][ ][ ] (dd/mm/yyyy)
End date [ ][ ] / [ ][ ] / [ ][ ][ ][ ] (dd/mm/yyyy)

17. ICU entry 1 [_] Yes 2 [_] No
If yes,
Entry date [ ][ ] / [ ][ ] / [ ][ ][ ][ ] (dd/mm/yyyy)
Exit date [ ][ ] / [ ][ ] / [ ][ ][ ][ ] (dd/mm/yyyy)

18. Hospital discharge date [ ][ ] / [ ][ ] / [ ][ ][ ][ ] (dd/mm/yyyy)

19. Final Diagnosis
Primary diagnosis
1 [_] Pneumonia 2[_] Asthma attack 3 [_] Bronchiolitis
4 [_] Others, mention :…………………………………

Secondary diagnosis
1 [_] Acute diarrhea 2 [_] Sepsis
3 [_] Underweight 4 [_] Other: ……………………………….

20. Discharge status*:* 1 [_] Alive 2[_] Death 3 [_] Discharge against medical advice

QUESTIONER – HOSPITAL BASED I-PADS

The Burden of Pneumonia In Indonesia Children And The Association With Vitamin D Deficiency: The Indonesian Pneumonia And Vitamin D Study (I-PADS)

Circle or complete answers that apply

PATIENT'S INFORMATION

1. Study ID ☐☐ / ☐☐☐☐ / ☐ - ☐☐☐ / I-PADS
2. Patient's name ____
3. Medical record number ☐☐☐☐☐☐☐☐☐☐☐☐☐☐☐☐
4. Gender 1. Male 2. Female
5. Address ____
____
6. Phone number ☐☐☐☐☐☐☐☐☐☐☐☐
7. Referral 1. Home 2. General practitioner/Paeditrician
3. Other hospital/Primary Health Care
8. Nutritional status Weight ☐☐☐☐ grams
Weight/Length ☐☐, ☐☐
9. Vaccination status, please check if up to date

☐	Age 0	Hep B 0	☐	Age 4	DTP-Hep B-Hib 3/Polio 3
☐	Age 1	BCG	☐	Age 9	Measles
☐	Age 2	DTP-Hep B-Hib 1/Polio 1	☐	Age 18	DTP-Hep B-Hib, polio Booster
☐	Age 3	DTP-Hep B-Hib 2/Polio 2	☐	Pneumococcal vaccine (i.e Prevenar)	

PARENTS' INFORMATION

10. Parents' age Mother _____ years
11. Parents' education (Please fill out based on the code)
    code: 1: Middle school or less 2: High school
    3: University 4: Other
    Mother ☐ Father ☐

12. Family income (please circle the correct answer)
    1. <Rp 500.000,00
    2. Rp 500.000-Rp1.000.000
    3. Rp 1.000.000-Rp2.500.000
    4. Rp 2.500.000-Rp 5.000.000
    5. >Rp 5.000.000

RISK FACTORS

At birth and early years

(Please circle the correct answer or complete the boxes)

13. Gestational age ☐☐ weeks + ☐☐ days
14. Birth weight ☐☐☐☐ grams
15. Congenital Heart Disease 1. Yes 2. No If yes, mention___________
16. Congenital syndrome 1. Yes 2. No If yes, mention___________

Crowdedness

(Please circle the correct answer or complete the boxes)

17. Number of people at home (excluding the child) ☐☐ people
18. Child care attendance 1. Yes 2. No

Early Months Feeding

(Please circle the correct answer or complete the boxes)

19. Exclusive breastfeeding 1. Yes 2. No
20. Age started solid food ☐☐ Months + ☐☐ weeks
21. Age when formula started ☐☐ Months + ☐☐ weeks
22. Who is the primary care giver of the baby?? (please circle the answer)

    1. Mother 2. Father 3. Other family member 4. Babysitter

    5. Others, mention_____________

Air Pollution

23. Do any family members currently smoke (in the last 12 months)? (Please put the number that applies)

22.1	Mother	☐	1. Not at all	4. Yes, at least monthly
22.2	Father	☐	2. Yes, daily	
22.3	Other family member *(living in the same roof with child)*	☐	3. Yes, at least weekly	

24 If yes, on average how many cigarettes do you smoke? (Please put the number that applies)

23.1	Mother	☐	1. <12units
23.2	Father	☐	2.12-24 units
23.3	Other family member *(living in the same roof with child)*	☐	3. >24 units

25 The main material for cooking (please circle the correct answer)

1. Electric	3. Kerosene	5. Charcoal
2. Gas	4. Wood	6. Other

Sun exposure

26 Do you usually take your child outside the house? (Please circle and complete answer that applies)

25.1	**On a weekday**	1. Yes, __________times/week	2. No
25.2	**On a weekend**	1. Yes,__________ times/week	2. No

27 At what time of the day do you usually take your child outside the house (Please tick the correct answer)

No	Hour	On Weekdays	On Weekend
27.1	Before 10.00am		
27.2	Between 10.00am – 16.00pm		
27.3	After 16.00 pm		

28 On average, how many hour do you take your child outside in a day (Please put the number that applies)

28.1 **On a weekday** ☐

28.2 **On a weekend** ☐

1. Less than 15 min
2. 15 min to 30 min
3. 31 min to 1 hour
4. 1 to 2 hours
5.2 to 3 hours
6. 4 to 5 hours
7. 5 to 6 hours

29 What does your child usually wear to go outside **<u>during a warm sunny day</u>**? (Please tick the correct answer)

29.1 Arms exposed

No	Arms	On Weekdays	On Weekend
1	Not exposed at all		
2	< 50% time exposed		
3	≥ 50% time exposed		
4	Always exposed		

29.2 Lower extremity exposed (thigh to heel))

No	Lower Extremity	On Weekdays	On Weekend
1	Not exposed at all		
2	< 50% time exposed		
3	≥ 50% time exposed		
4	Always exposed		

29.3 Sun Screen Usage

No	Usage	On Weekdays	On Weekend
1	Always		
2	< 50% of the time		
3	≥ 50% of the time		
4	Not at all		

29.4 Sunglasses Usage

No	Usage	On Weekdays	On Weekend
1	Always		
2	< 50% of the time		
3	≥ 50% of the time		
4	Not at all		

29.5 Umbrella Usage

No	Usage	On Weekdays	On Weekend
1	Always		
2	< 50% of the time		
3	≥ 50% of the time		
4	Not at all		

CRF – I-PADS BERBASIS RUMAH SAKIT

Beban Penyakit Pneumonia pada Anak-anak di Indonesia dan Hubungan dengan Defisiensi Vitamin D: Penelitian Indonesian Pneumonia and Vitamin D (I-PAD)

Petunjuk :Lingkari atau lengkapilah dengan jawaban yang sesuai

ID peserta ☐☐ / ☐☐☐☐ / ☐ - ☐☐☐ / I-PADS

Hospital
1: RSUD Wates
2: RSUD Kota Yogyakarta
3: RSUP Dr Sardjito

1. Nama pasien ____

2. Nomor Rekam Medis ☐☐☐☐☐☐☐☐☐☐☐☐☐☐☐☐
3. Jenis Kelamin 1. Laki-laki 2. Perempuan
4. Tanggal lahir ☐☐ / ☐☐ / ☐☐☐☐ (dd/mm/yyyy)
5. Berat Badan ☐☐ kg
6. Panjang Badan ☐☐☐ cm

Admisi IGD/ Poli (RS)–Diisi oleh dokter yang sedang bertugas
Petunjuk : Lengkapi jawaban dan lingkarilah jawaban yang sesuai

7. Tanggal admisi IGD/poli ☐☐ / ☐☐ / ☐☐☐☐ (dd/mm/yyyy)
8. Pukul admisi IGD/poli ☐☐ / ☐☐ WIB
9. Jalur admisi IGD/poli
   1. Poliklinik anak
   2. Instalasi Gawat Darurat (IGD)
10. Dokter yang bertanggung jawab ____

11. Gejala utama
    Lingkari yang sesuai
    (bisa lebih dari satu)
    1. Batuk
    2.Pilek
    3. Demam
    4.Tachypnea
    5. Mengi/wheeze
    6. Dyspnea
    7. Lainnya, _____

12. Tanda bahaya
    1. Tidak dapat minum/menyusu
    2. Stridor
    3. Lemah/ tidak sadarkan diri
    4. Sianosis
    5. Distress pernapasan berat
    6. Kejang
    7. Memuntahkan apapun
    8. Apnoea

13. Pemeriksaan fisik
    13.1 Respiration Rate (RR) ☐☐ / menit
    Respiration Rate (RR) hitungan 2 ☐☐ / menit
    (hitungan ke-2 dilakukan bila hitungan 1 meningkat)

13.2 Tarikan dinding dada ke dalam 1. Ya 2.Tidak
13.3 Pulse oximetry 1. Ya 2.Tidak
(tanpa menerima terapi oksigen) Hasil ☐☐☐%
Perhatian: Jangan lupa pasang kembali canule

14. Menerima bronchodilator hirup (inhaled) 1. Ya 2.Tidak
14.1 Jika ya, bagaimana respon setelah diberikan
Mengi 1. Ada 2.Tidak

15. Diagnosis awal
1. Pneumonia
2. Bronchiolitis
3. Asma
4. Other ___________________

16. Terapi antibiotic awal
1. Cotrimoxazole
2. Erythromycin
3. Cefotaxim
4. Penicillin
5. Ampicillin
6. Amoxicillin
7. Gentamycin
8. Amoxicillin clauvant
9. Ceftriaxone
10. Other,_______________
____
(dapat menyebutkan lebih dari 1)

ADMISI BANGSAL – diisi oleh tim peneliti
<u>Petunjuk :</u> Lingkarilah jawaban yang sesuai

Dilengkapi dalam 24 jam setelah admisi:

17. Admisi bangsal anak ☐☐ / ☐☐ / ☐☐☐☐ (dd/mm/yyyy)
18. Pukul ☐☐ / ☐☐ WIB
19. Inisial tim peneliti ☐☐☐

20. Tanda bahaya
1. Tidak dapat minum/menyusu
2. Stridor
3. Lemah/tidak sadarkan diri
4. Sianosis
5. Distress pernapasan berat
6. Kejang
7. Memuntahkan apapun
8. Apnoea

21. Pemeriksaan fisik
21.1 Respiration Rate (RR) ☐☐ / menit
Respiration Rate (RR) hitungan 2 ☐☐ / menit
(hitungan ke-2 dilakukan bila hitungan 1 meningkat)
21.2 Tarikan dinding dada ke dalam 1. Ya 2.Tidak
21.3 Pulse oximetry 1. Ya 2.Tidak
(tanpa menerima terapi oksigen) Hasil ☐☐☐%
Perhatian: Jangan lupa pasang kembali canule

22. Pemeriksaan darah
22.1 Darah rutin
Hemoglobin ☐☐ , ☐ g/dL
Neutrophil ☐☐ %
Jumlah trombosit ☐☐☐☐☐☐ /µL

Jumlah leukosit ☐☐☐☐☐☐ / µL

Limfosit ☐☐ %

Dilengkapi selama rawat inap:

22.2 Pemeriksaan CRP (jika dilakukan)

Kualitatif 1. Positif 2. Negatif

Kuantitatif ☐☐☐ mg/L

22.3 Kultur darah (jika dilakukan)

Tanggal ☐☐ / ☐☐ / ☐☐☐☐

Waktu pelaksanaan 1. Sebelum pemberian antibiotik

2. Setelah pemberian antibitok

Hasil kultur darah:

Negatif	1. Tidak	2. Ya
Streptococcus pneumonia	1. Tidak	2. Ya
Staphylococcus	1. Tidak	2. Ya
Pseudomonas	1. Tidak	2. Ya
Klebsiela pneumoniae	1. Tidak	2. Ya
Haemophylus influenza	1. Tidak	2. Ya
Lain-lain, sebutkan	______________________	

23. Pemeriksaan Radiologi ☐ Ya ☐ Tidak

Kesimpulan hasil pemeriksaaan pihak RS (site):

24. Copy foto rontgen thorax terambil ☐ Ya ☐ Tidak

*(**Perhatian:** Jangan lupa mengambil foto dari foto rontgen thorax)*

25. Terapi antibiotik

1. Cotrimoxazole
2. Erythromycin
3. Cefotaxim
4. Penicillin
5. Ampicillin
6. Amoxicillin
7. Gentamycin
8. Amoxicillin clauvant
9. Ceftriaxone
10. Other,__________

26. Apakah dilakukan penggantian antibiotic saat rawat inap?

1. Ya   2.Tidak

26.1 Jika ya, Antibiotik diganti menjadi:

1. Cotrimoxazole
2. Erythromycin
3. Cefotaxim
4. Penicillin
5. Ampicillin
6. Amoxicillin
7. Gentamycin
8. Amoxicillin clauvant
9. Ceftriaxone
10. Other,__________

____

(dapat menyebutkan lebih dari 1)

26.2 Perubahan disarankan pada hari ke_________

27. Oksigenasi   1.Ya   2.Tidak

27.1 Tanggal mulai ☐☐ / ☐☐ / ☐☐☐☐ (dd/mm/yyyy)

27.2 Tanggal berakhir ☐☐ / ☐☐ / ☐☐☐☐ (dd/mm/yyyy)

27.3 Metode (lingkari sesuai jawaban)   1. Nasal canule   2. Rebreathing mask   3. Non-rebreathing mask   4. Head box

28. Masuk ICU   1.Ya   2.Tidak

28.1 Tanggal mulai ☐☐ / ☐☐ / ☐☐☐☐ (dd/mm/yyyy)

28.2 Tanggal berakhir ☐☐ / ☐☐ / ☐☐☐☐ (dd/mm/yyyy)

KELUAR RUMAH SAKIT (RS) – Diisi oleh tim penelitian

Petunjuk: Lingkarilah jawaban yang sesuai

29. Tanggal keluar RS ☐☐ / ☐☐ / ☐☐☐☐ (dd/mm/yyyy)

30. Diagnosis akhir

1. Pneumonia
2. Bronchiolitis
3. Asthma
4. Other,___________

31. Status

1. Sembuh
2. Atas Permintaan Pasien
3. Meninggal dunia

KUESIONER – I-PADS BERBASIS RUMAH SAKIT

Beban Penyakit Pneumonia pada Anak-anak di Indonesia dan Hubungan dengan Defisiensi Vitamin D: Penelitian Indonesian Pneumonia and Vitamin D (I-PAD)

Lingkarilah atau lengkapilah dengan jawaban yang sesuai
INFORMASI PASIEN

1. ID peserta ☐☐ / ☐☐☐☐ / ☐ - ☐☐☐ / I-PADS
2. Nama peserta ____________________
3. Nomor Rekam Medis ☐☐☐☐☐☐☐☐☐☐☐☐☐☐☐☐
4. Jenis Kelamin 1. Laki-laki 2. Perempuan
5. Alamat ____________________
____
6. Nomor telepon ☐☐☐☐☐☐☐☐☐☐☐☐
7. Asal rujukan 1. Rumah 2.Dokter umum/ dokter anak
3. Rumah sakit/ puskesmas lainnya
8. Status nutrisi Berat badan ☐☐☐☐ kg
Panjang/tinggi badan ☐☐☐ cm
9. Status imunisasi, tolong beri tanda centang apabila sudah menerima (usia dalam bulan)

☐	Usia 0	Hep B 0	☐	Usia 4	DTP-Hep B-Hib 3/Polio 3
☐	Usia 1	BCG	☐	Usia 9	Campak
☐	Usia 2	DTP-Hep B-Hib 1/Polio 1	☐	Usia18	DTP-Hep B-Hib, polio Booster
☐	Usia 3	DTP-Hep B-Hib 2/Polio 2	☐	Vaksin Pneumococcal (i.e. Prevenar)	

INFOMASI ORANGTUA

10. Tanggal lahir Ibu ☐☐ / ☐☐ / ☐☐☐☐ (dd/mm/yyyy)
Usia ibu ☐☐ tahun
11. Pendidikan orang tua (isikan berdasarkan kode yang tersedia)
kode: 1: SMP atau dibawahnya 2: SMA/ sederajat
3: Universitas/ sederajat 4: Lainnya
Ibu ☐ Bapak ☐

12. Pemasukan keluarga (Lingkarilah jawaban yang sesuai)
    1. <Rp 500.000,00
    2. Rp 500.000-Rp1.000.000
    3. Rp 1.000.000-Rp2.500.000
    4. Rp 2.500.000-Rp 5.000.000
    5. >Rp 5.000.000

FAKTOR RISIKO

Saat kelahiran dan awal kelahiran

(lingkarilah jawaban yang sesuai atau lengkapi kotak yang tersedia)

13. Usia kehamilan ☐☐ minggu+ ☐☐ hari
14. Berat bayi lahir ☐☐☐☐ gram
15. Kelainan Jantung Bawaan 1. Ya 2.Tidak Jika ya, sebutkan___________
16. Sindrom bawaan 1. Ya 2.Tidak Jika ya, sebutkan___________

Kepadatan

(lingkarilah jawaban yang sesuai atau lengkapi kotak yang tersedia)

17. Jumlah orang yang tinggal serumah dengan anak (tidak termasuk anak) ☐☐orang
18. Apakah anak Anda juga diasuh di Tempat Penitipan Anak (TPA)? (silakan lingkari jawaban yang sesuai)
    1. Ya 2.Tidak

Kebiasaan makan pada awal kehidupan

(lingkarilah jawaban yang sesuai atau lengkapi kotak yang tersedia)

19. ASI eksklusif 6 bulan 1. Ya 2.Tidak
20. Usia mulai makanan padat ☐☐ bulan + ☐☐ minggu
21. Usia mulai susu formula ☐☐ bulan + ☐☐ minggu
22. Siapakah yang menjadi pengasuh utama anak? (silakan lingkari jawaban yang sesuai)
    1. Ibu 2. Ayah 3. Keluarga lainnya 4. Pengasuh anak/babysitter
    5. Lainnya, sebutkan_____________

Polusi udara

23. Adakah anggota keluarga yang merokok (dalam kurun waktu 12 bulan terakhir)? (tuliskan nomor yang sesuai pada kotak yang tersedia dan (-) jika tidak)
    23.1 Ibu ☐ 1. Tidak sama sekali 4. Ya, minimal per bulan
    23.2 Bapak ☐ 2. Ya, setiap hari
    23.3 Anggota keluarga lain ☐ 3. Ya, minimal per minggu
    (yang tinggal satu rumah dengan anak)

24. Jikaya, rata-rata berapa banyak batang rokok yang dikonsumsi? (tuliskan nomor yang sesuai pada kotak yang tersedia dan (-) jika tidak)
    24.1 Ibu ☐ 1. ≤12batang
    24.2 Bapak ☐ 2.13-24 batang
    24.3 Anggota keluarga lain ☐ 3. >24 batang
    (yang tinggal satu rumah dengan anak)

25. Apakah bahan bakar utama yang digunakan dirumah untuk memasak? (lingkarilah jawaban yang sesuai)

1. Listrik 3. Minyak tanah 5. Arang
2. Gas 4. Kayu 6. Lainnya

Paparan sinar matahari

26. Apakah Anda biasa membawa anak Anda keluar rumah? (lingkarilah dan lengkapilah jawaban yang sesuai)

26.1 **Pada hari kerja** 1. Ya, __________kali/minggu 2. Tidak
26.2 **Pada akhir pekan** 1. Ya,__________ kali/minggu 2. Tidak

27. Kapan Anda biasanya membawa anak keluar rumah? (berikan tanda centang pada jawaban yang sesuai)

**No**	**Jam**	**Pada hari Kerja**	**Pada akhir pekan**
27.1	Sebelum jam 10.00		
27.2	Antara jam 10.00 – 16.00		
27.3	Setelah jam 16.00		

28. Rata-rata, berapa lama Anda membawa anak Anda keluar ruangan? (Isikan nomor yang sesuai dengan jawaban Anda)

28.1 **Pada hari kerja** ☐
28.2 **Pada akhir pekan** ☐

1. Kurang dari 15 menit 4. 2 jam 6. 4 jam
2. 15 menit - 30 menit 5. 3 jam 7. 5 jam
3. 31 menit - 1 jam

29. Bagaimanakah kebiasaan berpakaian anak selama terpapar sinar matahari (beri tanda centang pada jawaban yang sesuai)

29.1 Terekspos bagian lengan

**No**	**Bagian Lengan**	**Pada hari Kerja**	**Pada akhir pekan**
1	Tidak terpapar sama sekali		
2	< 50% waktu terpapar		
3	≥ 50% waktu terpapar		
4	Selalu terpapar		

29.2 Terekspos bagian esktremitas bawah (paha hingga tumit)

**No**	**Bagian esktremitas bawah**	**Pada hari Kerja**	**Pada akhir pekan**
1	Tidak terpapar sama sekali		
2	< 50% waktu terpapar		
3	≥ 50% waktu terpapar		
4	Selalu terpapar		

29.3 Menggunakan tabir surya

No	Penggunaan	Pada hari Kerja	Pada akhir pekan
1	Selalu menggunakan		
2	Menggunakan < 50% waktu terpapar		
3	Menggunakan ≥ 50% waktu terpapar		
4	Tidak menggunakan sama sekali		

29.4 Menggunakan kacamata

No	Penggunaan	Pada hari Kerja	Pada akhir pekan
1	Selalu menggunakan		
2	Menggunakan < 50% waktu terpapar		
3	Menggunakan ≥ 50% waktu terpapar		
4	Tidak menggunakan sama sekali		

29.5 Menggunakan payung

No	Penggunaan	Pada hari Kerja	Pada akhir pekan
1	Selalu menggunakan		
2	Menggunakan < 50% waktu terpapar		
3	Menggunakan ≥ 50% waktu terpapar		
4	Tidak menggunakan sama sekali		
